# The association between sedentary behavior and low back pain in adults: a systematic review and meta-analysis of longitudinal studies

**DOI:** 10.7717/peerj.13127

**Published:** 2022-03-28

**Authors:** Hosam Alzahrani, Mansour Abdullah Alshehri, Msaad Alzhrani, Yasir S. Alshehri, Wesam Saleh A. Al Attar

**Affiliations:** 1 Department of Physical Therapy, College of Applied Medical Sciences, Taif University, Taif, Saudi Arabia; 2 Department of Physiotherapy, College of Applied Medical Sciences, Umm Al Qura University, Mecca, Saudi Arabia; 3 NHMRC Centre of Clinical Research Excellence in Spinal Pain, Injury and Health, School of Health and Rehabilitation Sciences, University of Queensland, Brisbane, Australia; 4 Department of Physical Therapy and Health Rehabilitation, College of Applied Medical Sciences, Majmaah University, Majmaah, Saudi Arabia; 5 Department of Physical Therapy, College of Medical Rehabilitation Sciences, Taibah University, Madinah, Saudi Arabia; 6 Department of Sport, Exercise and Health, Faculty of Medicine, University of Basel, Basel, Switzerland; 7 Discipline of Exercise and Sport Science, Faculty of Medicine and Health Sciences, The University of Sydney, Sydney, Australia

**Keywords:** Low back pain, Sedentary behavior, Risk factor, Meta-analysis

## Abstract

**Background:**

Low back pain (LBP) is a common musculoskeletal problem globally. While spending a longer time in sedentary behaviors is linked to several health problems; the quantitative association between different amounts of sedentary time and LBP is still unknown. This study aims to systematically review studies that examined the association between sedentary behavior and LBP development and LBP-related outcomes.

**Methods:**

This systematic review and meta-analysis retrieved journal articles published from inception to March 2020 and were obtained by searching bibliographical databases. We included longitudinal study designs, including adult (aged ≥18) individuals with nonspecific LBP, and reporting estimates of the association between sedentary behavior and LBP development and LBP-related outcomes (*i.e*., pain intensity and disability).

**Results:**

Sixteen longitudinal studies with 100,002 participants were included in this review (eight studies included in quantitative syntheses with 83,111 participants). The results of meta-analyses showed that a sedentary time of 3–<6 (Odds ratio (OR) 0.95, 95% CI [0.85–1.07]), 6–8 (OR 0.95, 95% CI [0.88–1.02]), and >8 (OR 0.92, 95% CI [0.85–1.00]) hours per day (h/d) was not associated with LBP development. A sedentary time of ≥3 h/d was associated with poor LBP-related disability (OR 1.24, 95% CI [1.02–1.51]), but not with pain intensity.

**Conclusion:**

A meta-analyses of longitudinal studies indicated that sedentary behavior of different durations was not associated with LBP development. However, the results showed that sedentary behavior ≥3 h/d was associated with worse LBP-related disability. These conclusions are tentative as the evidence was derived from mostly fair-quality studies using subjective measures of sedentary behavior.

**Systematic review registration:**

PROSPERO (registration number CRD42018107078).

## Introduction

Low back pain (LBP) is a common musculoskeletal condition that affects most people at some point in their lifetime (Walker, 2000). Slow recovery in some individuals with LBP can affect the individual’s physical and psychosocial function and increase the socioeconomic burden (Hoy et al., 2012; Valat, 2005; van Tulder, Koes & Bombardier, 2002). LBP was also the leading cause of years lived with disability in 2017 (Wu et al., 2020). Most cases can be triaged as nonspecific LBP where a specific etiology has not been determined (van Tulder et al., 2006).

Sedentary behavior is defined as “any waking behavior characterized by an energy expenditure of ≤1.5 metabolic equivalents (MET) while in the sitting or reclining posture” (Barnes et al., 2012; Owen et al., 2010). As such, sedentary behavior includes a broad range of behaviors such as watching television, using a computer, playing video games and sitting at work. A previous study has shown that adults spend more than half of their waking hours in sedentary behaviors (Matthews et al., 2008). Spending a longer time in sedentary behaviors is linked to several health problems. For example, sedentary behavior has been shown to be associated with cardiovascular disease, diabetes, cancer and mortality from all causes (Edwardson et al., 2012; Katzmarzyk et al., 2009; Thorp et al., 2011).

The link between sedentary behavior and some other health outcomes such as LBP, however, remains uncertain. Some studies found that sitting time was associated with LBP (Gupta et al., 2015; Omokhodion & Sanya, 2003), but other studies did not (Macfarlane et al., 1997; Xu, Bach & Orhede, 1997). The potential mechanisms of this association between sedentary behavior and LBP may be due to the biomechanical disadvantages of prolonged sitting on the lumbar spine such as decreased lower-back muscle strength (Kong, 2010) and increased lumbar spine stiffness (Beach et al., 2005). Furthermore, the sedentary time has been shown to be associated with adverse psychological health which might consequently contribute to LBP (Hamer, Coombs & Stamatakis, 2014; Pinheiro et al., 2016; Teychenne, Costigan & Parker, 2015). To the best of our knowledge, three reviews (without meta-analyses) (Chen et al., 2009; Hartvigsen et al., 2000; Lis et al., 2007) were identified with a similar topic and no association between sedentary behavior and LBP was found. However, two reviews dealt with sitting at work only (Hartvigsen et al., 2000; Lis et al., 2007), and the third review included 15 studies (10 prospective cohorts and five case–controls) published only up to 2006, and also included children in addition to adults (Chen et al., 2009). The quantitative association between different amounts of sedentary time and LBP, independent of other risk factors for LBP, is still unknown. Therefore, no consensus recommendations have been issued regarding limits on the amount of sedentary time to optimize LBP prevention. To address this knowledge gap, we performed a pooled analysis of longitudinal studies using a meta-analytical approach to examine the association between sedentary time and LBP development and LBP-related outcomes.

## Methods

### Design

This study was a systematic review of longitudinal studies. The protocol of this review was developed and pre-registered with the International Prospective Register of Systematic Reviews (PROSPERO; registration number CRD42018107078). This review was conducted following the Preferred Reporting Items for Systematic Reviews and Meta-Analyses (PRISMA) guidelines (Moher et al., 2009).

### Identification and selection of studies

Journal articles published from inception to March 2020 were obtained by searching the following bibliographical databases: Medline *via* OvidSP (1946–present), CINAHL *via* EBSCOhost (1981–present), Scopus, EMBASE (1947–present) and Web of Science. The search strategy included terms related to sedentary behavior and LBP. We also screened the reference lists of the identified papers and systematic reviews for additional articles. Table S1 provides a full electronic search strategy.

Two independent reviewers (HA, MAA) searched the information sources. Both reviewers screened the identified articles independently using the registered protocol and made decisions about inclusion according to the inclusion and exclusion criteria. The disagreements between both reviewers were resolved by consensus or third reviewer (WSA). Titles were screened initially, then abstracts, followed by full-text articles.

**Figure 1:**
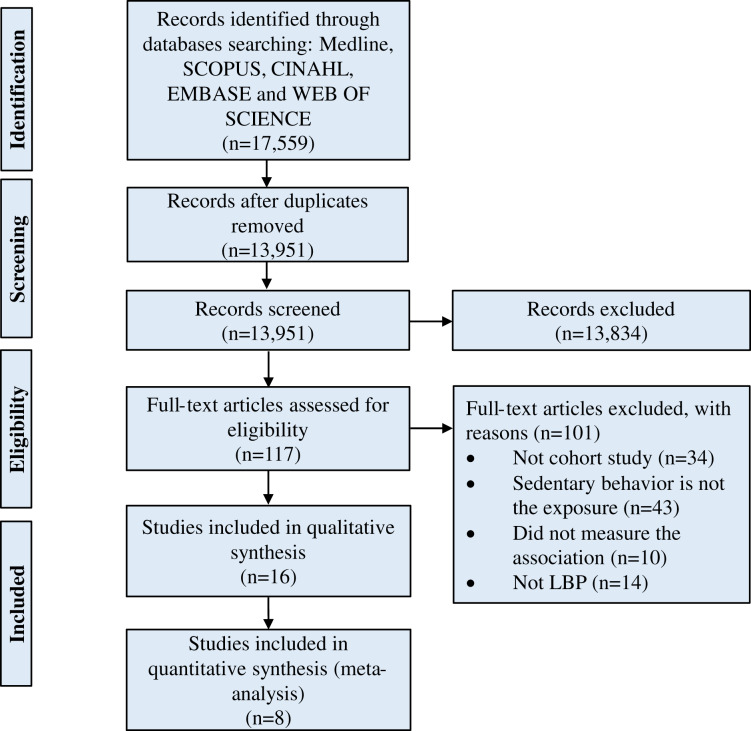
Flow of studies through the review.

Following the discussion between both reviewers, an article was considered potentially relevant and its full text was reviewed if it could not be unequivocally excluded based on its title and abstract (Moher et al., 2009; Tacconelli, 2010). All full-text articles that were included based on title or abstract were then screened. The number of included and excluded articles at different phases was recorded as recommended (Hicks, 2009) and shown in a PRISMA flowchart (Fig. 1).

### Eligibility criteria

The studies were included if they met the following criteria:

- Longitudinal study design.
- The study included adult (aged ≥18) individuals with nonspecific LBP.
- The study reported estimates of the association between sedentary behavior (exposure) and LBP development (outcome). This review also considered studies that reported estimates of the association between sedentary behavior and LBP-related outcomes (*i.e*., pain intensity or disability).

Studies were excluded if they were cross-sectional or case-control studies or used an experimental design, were in languages other than English, or they included participants with LBP attributed to a specific cause such as serious pathology, herniated intervertebral disk, osteoporosis, fracture, neurological compromise or cancer.

### Data extraction

Two independent reviewers (HA, MAA) extracted data using a specific form tailored to the requirements of this review. Disagreements between the two reviewers regarding extracted data were resolved through discussion. The extracted data included the following: main author, study design, study population, sample size, participant characteristics, LBP prevalence, sedentary behavior types and measurements, and main findings. If potentially relevant data were missing from the eligible studies, the main or corresponding author of these studies was contacted.

### Data synthesis and analysis

The analyses were conducted to investigate the associations between different durations of sedentary behaviors and LBP development and LBP-related outcomes. All the variables of sedentary behavior extracted from included studies were classified into groups defined around tertiles. This method of classification was followed in a previous published review (Alzahrani et al., 2019). Three different durations of sedentary behavior were included in the analyses: (a) sedentary duration of 3–<6 h/d, (b) sedentary duration of 6–8 h/d, and (c) sedentary duration above 8 h/d. We also conducted meta-analyses to examine the association between sedentary behavior (≥3 h/d) and LBP-related outcomes (*i.e*., pain intensity and disability).

When a study used a range to describe an exposure (*e.g*., 0–2 h/d sitting), we used a point estimate, which was the midpoint of the range. When a study used an unbounded or open category to describe an exposure, we assumed that the size of this category is the same as the closest equivalent exposure category and then calculate the median. For example, if a study categorized sitting as 0–2 h/d, 2–4 h/d and >4 h/d, we assumed that the last exposure group is 4–6 h/d and uses the median value of 5 h/d. If a study used only one open category *vs* the reference (*e.g*., > or <3 h/d *vs* none), we assumed the size of that category is 3 ± 1.5 = 4.5 or 1.5 h/d. These rules were reported in previous published reviews (Alzahrani et al., 2019; Kodama et al., 2013). Furthermore, one of the included studies (Juul-Kristensen et al., 2004) reported only the percentage of time spent working on computer per day in Denmark; however, the analyses were based on employees working 32–41 h/w (around 8 h/d) as mentioned by the author in another published study that used the same data (Jensen et al., 2002).

We pooled and synthesized the data using the Review Manager program (RevMan) (RevMan The Cochrane Collaboration, 2014). Of the eight studies included in the meta-analyses, seven studies reported ORs and only one study reported hazard ratio (HR). Therefore, in this review, OR was used as the common measure of association for the meta-analyses of studies. The fully adjusted odds ratio (OR) was obtained by comparing different durations spent in sedentary behavior with the lowest duration (reference category). The data were pooled and calculated as the inverse variance weighted mean of the logarithms of OR with their 95% CI (confidence interval) (Higgins & Green, 2011). For the study that used different measure in measuring the association between the variables (*i.e*., HR), we interpreted it as OR estimate as this study did not provide information to convert HR to OR (Behrens & Leitzmann, 2013; Clair et al., 2015). For studies that reported separate results for different groups (*e.g*., male and female, or different age groups), we included all groups in the meta-analysis because these different groups were independent of each other.

We evaluated statistical heterogeneity among studies using the Higgins *I*^*2*^ statistic test (Higgins & Thompson, 2002), and a value of *I*^*2*^ bigger than 50% was considered to indicate large heterogeneity (Higgins & Thompson, 2002; Higgins et al., 2003). A random-effect model was employed when there was a large heterogeneity; otherwise, a fixed-effect model was utilized (Higgins & Green, 2011). The results were considered statistically significant when the *P-*value was less than 0.05, or when the 95% CI about the OR did not cross 1. We could not assess publication bias using Egger’s regression test and funnel plots (Egger et al., 1997) because of the small number of studies included in a single meta-analysis (<10 studies) (Sterne et al., 2011).

We could not conduct a sensitivity analysis restricted to high-quality (good) studies (based on the National Institutes of Health’s Quality Assessment Tool) to examine the robustness of the results owing to the very small number of the included studies assessed as high quality. A sensitivity analysis was also conducted by using different cut-offs of the sedentary behavior duration (>2, >4, >6 and >8 h/d) to examine whether using different cut-offs change the results of the meta-analyses.

### Assessment of study quality

The methodological quality of the included studies was evaluated using the National Institutes of Health’s Quality Assessment Tool (Table S2) (National Institutes of Health, 2014). The assessment was conducted independently by two reviewers. Disagreements were resolved by mutual consent. Fourteen criteria were used to evaluate the methodological quality of the studies. The percentages of the 14 items scored with “yes” were calculated. The studies were classified as good (75–100%), fair (25–75%) or poor (0–25%); this method was used in a previously published study (San Giorgi et al., 2016).

## Results

### Search results

A total of 17,559 studies were retrieved, and after removing duplicates, 13,951 studies remained. Following the screening of titles and abstracts of these studies, 117 studies were eligible for assessment by full-text paper. Of 117 studies, 16 studies (Amorim et al., 2017; Andersen, Haahr & Frost, 2007; Balling et al., 2019; Harkness et al., 2003; Hartvigsen et al., 2001; Hartvigsen & Christensen, 2007; Hestbaek et al., 2005; Hussain et al., 2016; Juul-Kristensen et al., 2004; Kopec, Sayre & Esdaile, 2004; Lunde et al., 2017; Macfarlane et al., 1997; Matsudaira et al., 2012; Shiri et al., 2019; Venseth, 2014; Yip, 2004) fulfilled the inclusion criteria for qualitative syntheses, and eight (Balling et al., 2019; Harkness et al., 2003; Hussain et al., 2016; Juul-Kristensen et al., 2004; Macfarlane et al., 1997; Matsudaira et al., 2012; Shiri et al., 2019; Venseth, 2014) of these studies were included in the quantitative syntheses (Table S3). The remaining studies (*n* = 8) were excluded from quantitative syntheses because they specified no time of sedentary behavior (Amorim et al., 2017; Hestbaek et al., 2005), did not consider adjustment for potential confounding factors (Yip, 2004), used different units of measurement (*i.e*., sitting time/hour) (Andersen, Haahr & Frost, 2007) or used a different reference category (Hartvigsen et al., 2001; Hartvigsen & Christensen, 2007; Kopec, Sayre & Esdaile, 2004; Lunde et al., 2017). Figure 1 shows the flow of studies through the review.

### Characteristics of included studies

Sixteen longitudinal studies (Amorim et al., 2017; Andersen, Haahr & Frost, 2007; Balling et al., 2019; Harkness et al., 2003; Hartvigsen et al., 2001; Hartvigsen & Christensen, 2007; Hestbaek et al., 2005; Hussain et al., 2016; Juul-Kristensen et al., 2004; Kopec, Sayre & Esdaile, 2004; Lunde et al., 2017; Macfarlane et al., 1997; Matsudaira et al., 2012; Shiri et al., 2019; Venseth, 2014; Yip, 2004) with 100,002 participants (48.3% female) were included in the review. The mean prevalence of LBP was 13.3%. These studies were conducted in different countries, and many studies were conducted in Denmark (*n* = 6). The included studies involved a broad range of sedentary behaviors including television viewing, working on computer and desk work (office work) and total sitting. Table 1 shows the characteristics of the included studies.

**Table 1:** The characteristics of the included studies.

Study	Country	Population	Sample size (cases/total)	Age, mean (SD)	Sex, female (%)	Follow-up duration	Date of data collection	Sedentary behavior measure
Amorim et al. (2017)	Spain	Monozygotic (MZ) and dizygotic (DZ) adult twins	245/1,098	53.7 (7.3)	47.4	NA	2009–2013	Self-administered questionnaire
Andersen, Haahr & Frost (2007)	Denmark	Workers from industrial and service companies	160/1,513	NA	NA	2 years	NA	Self-administered questionnaire
Balling et al. (2019)	Denmark	National population	1,796/46,826	48.4 (15.4)	61.5	7.4 years	2007–2015	Long International Physical Activity Questionnaire (IPAQ-L)
Harkness et al. (2003)	United Kingdom	Newly employed workers from 12 diverse occupational groups	200/1,081	Median = 23, Interquartile range = 21–28	36	2 years	NA	Self-administered questionnaire
Hartvigsen & Christensen (2007)	Denmark	Twin individuals (monozygotic and same-sexed dizygotic twin pairs)	172/1,387	77.0 (NA)	52	2 years	2001–2003	Self-administered questionnaire
Hartvigsen et al. (2001)	Denmark	People living in a small Danish town	48/1,163	40.4 (NA)	53	5 years	NA	Self-administered questionnaire
Hestbaek et al. (2005)	Denmark	Military conscripts at 15 locations	345/985	20.57 (2.16)	4	3 months	2000	Self-administered questionnaire
Hussain et al. (2016)	Australia	National population	4144/5,058	NA	56	15 years	1999–2014	Active Australia Survey
Juul-Kristensen et al. (2004)	Denmark	Office workers	592/2,576	NA	61	1.9 years	1999–2000	Self-administered questionnaire
Kopec, Sayre & Esdaile (2004)	Canada	Canadian National Population	855/10,007	NA	55.3	2 years	1996–1997	Self-administered questionnaire
Lunde et al. (2017)	Norway	Construction and healthcare workers	124/594	NA	NA	6 months	2014	ActiGraph GT3X accelerometer
Macfarlane et al. (1997)	United Kingdom	Convenient population registered in two general practices in south Manchester area	247/784	Median = 38 Range = 18–75	61	1 year	NA	Self-administered questionnaire
Matsudaira et al. (2012)	Japan	Office workers, nurses, sales/marketing personnel, and manufacturing engineers	308/836	44.2 (10.2)	11.6	2 years	NA	Self-administered questionnaire
Shiri et al. (2019)	Finland	National population	1,226/3,505	NA	52.7	11 years	2000–2011	Self-administered questionnaire
Venseth (2014)	Norway	National population	2,782/22,445	45.5 (NA)	52.5	10 years	1995–2006	Self-administered questionnaire
Yip (2004)	Hong Kong	Nurses	56/144	31.10 (NA)	85.5	1 year	2001–2002	Self-administered questionnaire

**Note:**

NA, Not Applicable.

Eight longitudinal studies with 83,111 participants (11,295 participants diagnosed with LBP at follow-ups) were included in the quantitative syntheses. These studies were adjusted for age and sex. Other potential confounding factors were also considered in less than 50% of studies such as mental status, psychosocial status, body mass index (BMI), education, smoking status, dietary guideline index, ergonomics, previous episodes of LBP, occupation, physical activity, occupational activities and other types of activities (Table S3).

### Quality

None of the included studies met all criteria of the quality assessment score (Table S4). Five (Andersen, Haahr & Frost, 2007; Harkness et al., 2003; Juul-Kristensen et al., 2004; Kopec, Sayre & Esdaile, 2004; Matsudaira et al., 2012) of 16 studies were rated as good quality using the National Institutes of Health’s Quality Assessment Tool. The mean score of the quality assessment was 9.6 out of 14 for the included studies (range, 8–12). Most studies (*n* = 14) failed to blind outcome assessors to the exposure status of participants. Three of the eight studies included in the meta-analyses were good quality (Harkness et al., 2003; Juul-Kristensen et al., 2004; Matsudaira et al., 2012).

### Sedentary behavior measurements

All included studies (*n* = 15), except one, used self-administered questionnaires to assess sedentary behaviors. The only study that used an objective measure of sedentary behavior (ActiGraph GT3X accelerometer) was Lunde et al. (2017). Of the eight studies included in the meta-analyses, five studies assessed total time spent sitting (Balling et al., 2019; Harkness et al., 2003; Macfarlane et al., 1997; Shiri et al., 2019; Venseth, 2014), two studies assessed sitting time while working on computer and desk work (office work) (Juul-Kristensen et al., 2004; Matsudaira et al., 2012), and one study assessed sitting time while viewing television (Hussain et al., 2016). Different durations of sedentary behavior were assessed in eight studies (Table S5).

### Association between sedentary behavior and LBP development

#### Sedentary duration of 3–<6 h/d *vs* lowest

**Figure 2:**
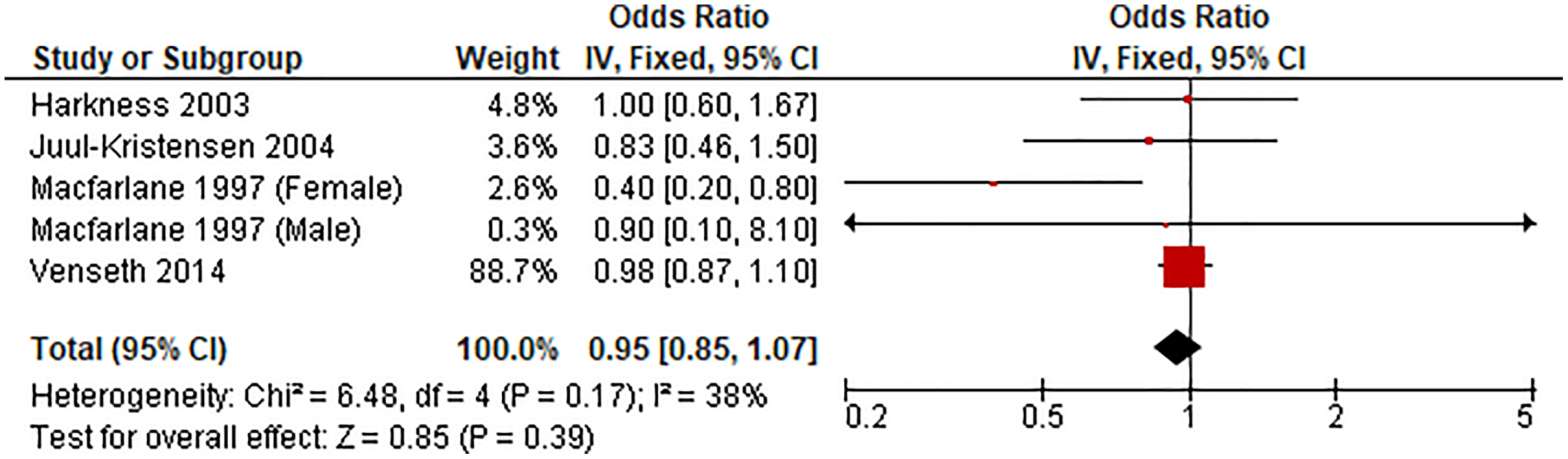
Association between sedentary behavior 3–<6 *vs* lowest and low back pain.

Figure 2 shows the pooled estimates of four studies (five comparisons) for the odds of LBP associated with sedentary time of 3–<6 h/d. Compared with the lowest sedentary time category, sedentary duration of 3–<6 was unassociated with LBP (OR 0.95, 95% CI [0.85–1.07], *P* = 0.39, *I*^*2*^ = 38%).

#### Sedentary duration of 6–8 h/d *vs* lowest

**Figure 3:**
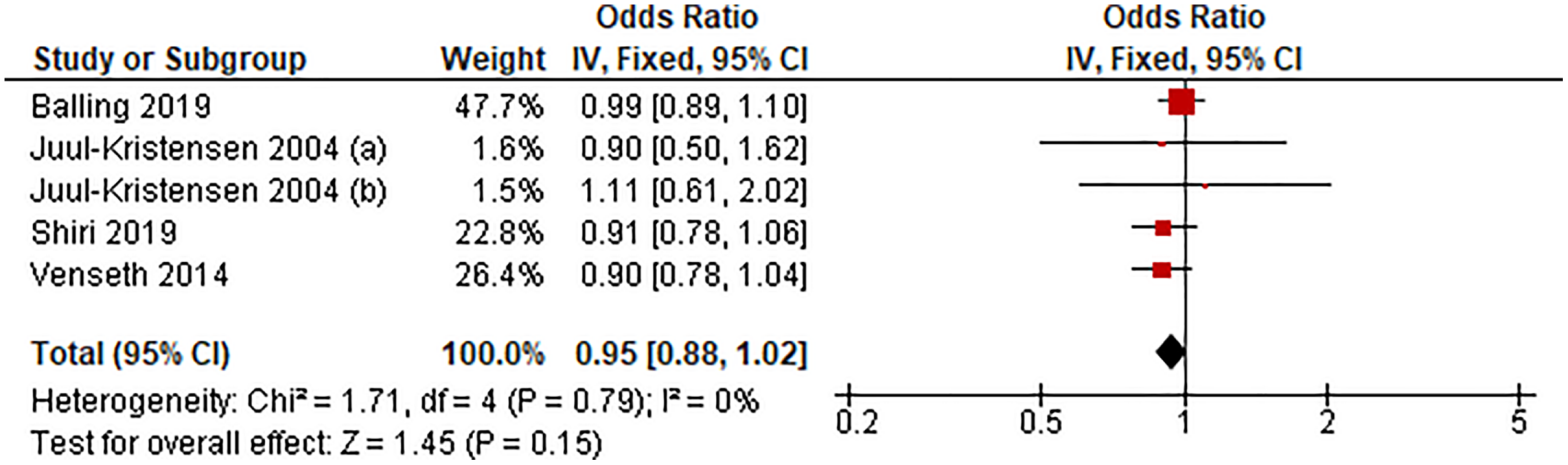
Association between sedentary behavior 6–8 h/d *vs* lowest and low back pain.

Figure 3 shows the pooled estimates of four studies (five comparisons) for the odds of LBP associated with sedentary time of 6–8 h/d. Compared with the lowest sedentary time category, sedentary duration of 6–8 h/d was unassociated with LBP (OR 0.95, 95% CI [0.88–1.02], *P* = 0.15, *I*^*2*^ = 0%).

#### Sedentary duration of >8 h/d *vs* lowest

**Figure 4:**
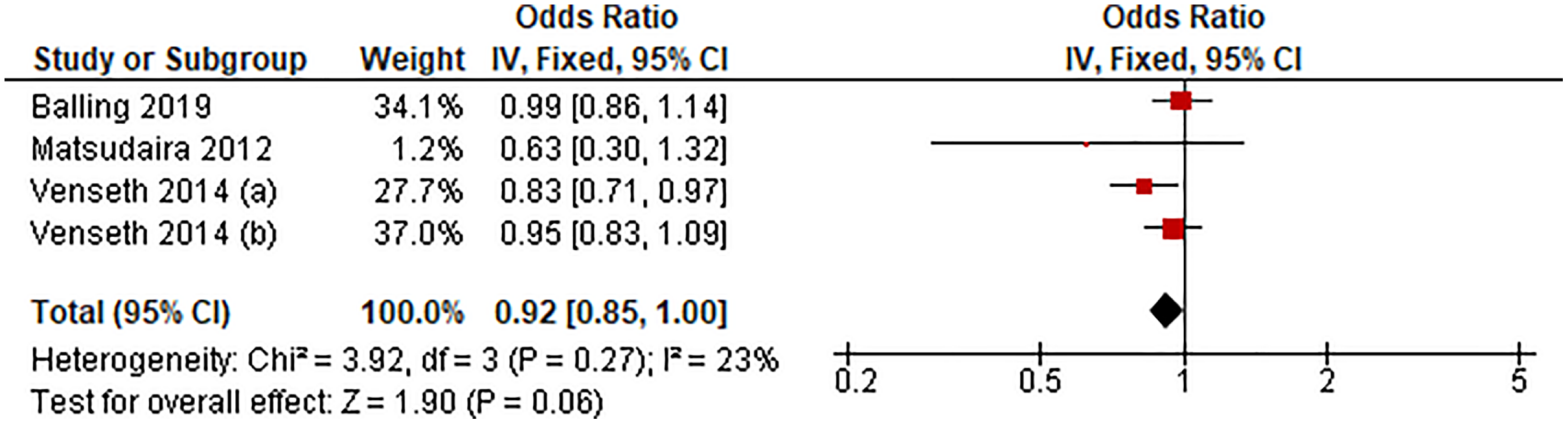
Association between sedentary behavior >8 h/d *vs* lowest and low back pain.

Figure 4 shows the pooled estimates of three studies (four comparisons) for the odds of LBP associated with sedentary time exceeding 8 h/d. Compared with the lowest sedentary time category, sedentary duration exceeding 8 h/d was unassociated with LBP (OR 0.92, 95% CI [0.85–1.00], *P* = 0.06, *I*^*2*^ = 23%).

### Association between sedentary behavior and LBP-related outcomes

**Figure 5:**
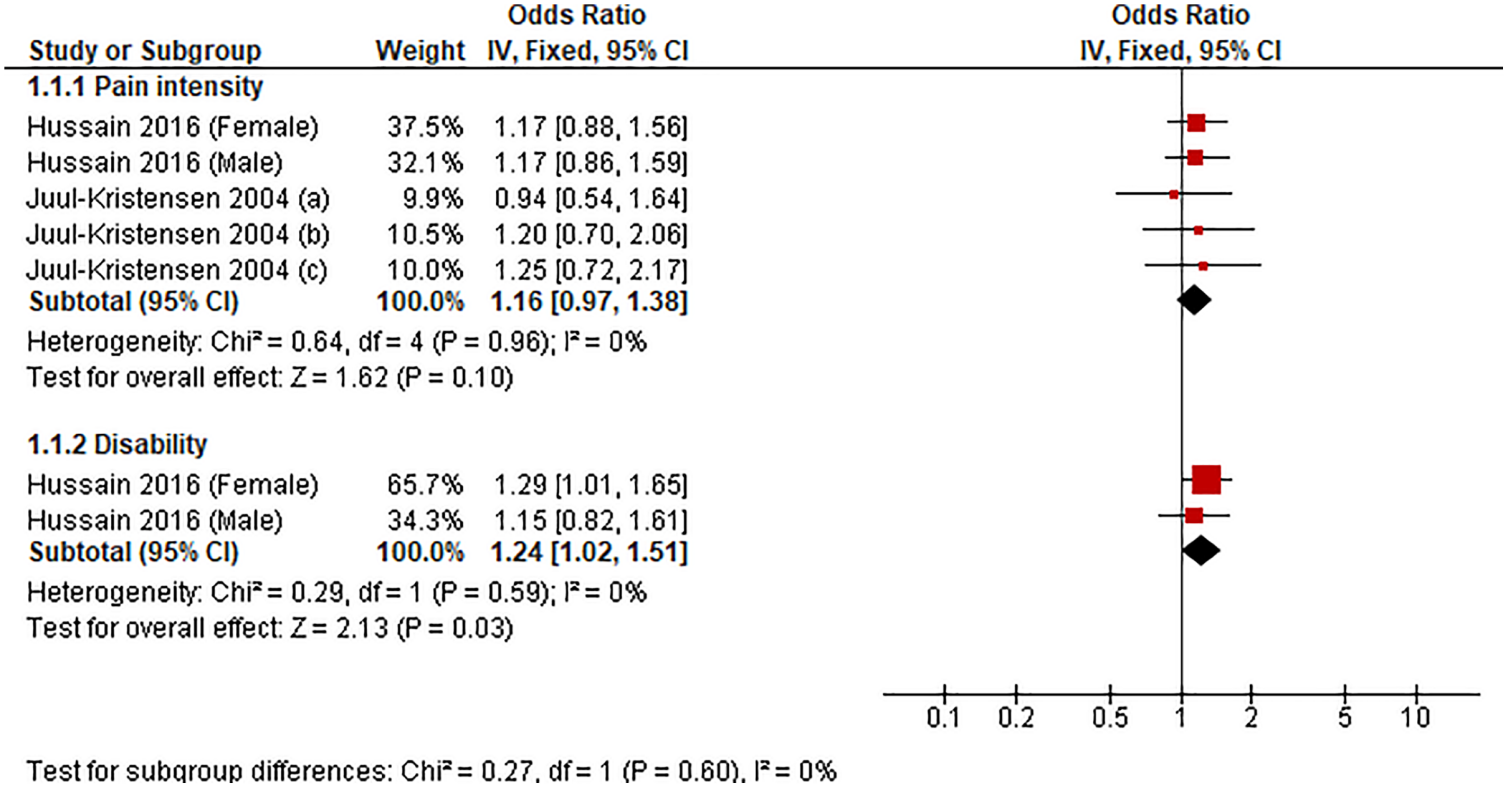
Association between sedentary behavior ≥3 h/d *vs* lowest and low back pain-related outcomes.

We could conduct a meta-analysis of two studies (seven comparisons) investigating the association between sedentary duration of ≥3 h/d, and LBP-related outcomes (*i.e*., pain intensity and disability) (Fig. 5). Compared with the lowest sedentary time category, sedentary behavior of ≥3 h/d was associated with disability (OR 1.24, 95% CI [1.02–1.51], *P* = 0.03, *I*^*2*^ = 0%) but not with pain intensity (OR 1.16, 95% CI [0.97–1.38], *P* = 0.10, *I*^*2*^ = 0%).

### Sensitivity analyses

The sensitivity analyses of using different cut-offs of the sedentary behavior durations showed that a sedentary time of >2 (OR 0.94, 95% CI [0.89–0.99]), >4 (OR 0.94, 95% CI [0.90–0.99]), and >6 (OR 0.94, 95% CI [0.89–0.99]) h/d was associated with a lower prevalence of LBP (Figs. S1–S3). However, no association was found with sedentary time >8 h/d (OR 0.92, 95% CI [0.85–1.00]) (Fig. S4).

## Discussion

This review determined the association between sedentary behavior and LBP development and LBP-related outcomes. The findings of this review indicated that the sedentary duration (3–<6, 6–8 or >8 h/d) was not associated with LBP development. Furthermore, sedentary behavior has been shown to be associated with LBP disability but not pain intensity, with a sitting duration ≥3 h/d leading to worse LBP-related disability.

To our knowledge, this is the first meta-analysis to investigate the association between different amounts of sedentary behavior and LBP. In this review, our quantitative analyses showed that the sedentary duration for 3–<6, 6–8 or >8 h/d was not associated with LBP. This finding agrees with previous reviews that suggested that sedentary behavior was unassociated with LBP (Chen et al., 2009; Hartvigsen et al., 2000; Lis et al., 2007). Some studies have shown, however, that sitting for a long duration without intermittent break increases intradiscal pressure and stiffness of the lumbar spine, and decreases the strength of the lower back muscles, which might consequently contribute to LBP (Billy, Lemieux & Chow, 2014; Hussain et al., 2016; Kong, 2010; Beach et al., 2005). These results differ from ours probably because some participants accumulate a large amount of moderate-to-vigorous physical activity in addition to too much sitting during the day. Engaging in moderate-intensity physical activity has a protective role against LBP, supporting the opinion that physical activity strengthens the back muscles (Alzahrani et al., 2019). Moreover, sedentary behavior can lead to adverse psychological health which might consequently contribute to LBP; however, engaging in physical activity can counteract the negative impact of sedentary behavior on psychological health (Blough & Loprinzi, 2018; Hamer, Coombs & Stamatakis, 2014; Liao et al., 2016; Pinheiro et al., 2016; Teychenne, Costigan & Parker, 2015). Therefore, time spent on sedentary behavior should not be analyzed in isolation from the remaining behaviors (*i.e*., physical activity and sleep) that compose the entire 24-h day as all behaviors are necessarily related to each other (Dumuid et al., 2020). As stated by Shanahan & Flaherty (2001), “time devoted to one domain of activity takes on full meaning only when viewed in terms of its functional relation to time spent in other domains”. Future research should investigate the joint association between the combination of time spent in physical activity, sedentary behavior and sleep and LBP, which reflects the daily exposure of individuals to all types of daily lifestyle behaviors.

In this review, we additionally investigated whether sedentary duration for ≥3 h was associated with LBP-related outcomes (*i.e*., pain intensity and disability). The results of the quantitative analyses of two longitudinal studies revealed that sedentary duration ≥3 h/d was associated with LBP-related disability but not pain intensity. Sedentary duration ≥3 h/d can increase LBP-related disability by 24%, and this probably occurs due to the misinterpretation of patients with nonspecific LBP of their case as a serious injury, which contributes to developing fears of movement and subsequent avoidance of movements, leading to disability (Swinkels-Meewisse et al., 2003; Vlaeyen et al., 1995).

Eight longitudinal studies with 83,111 participants (11,295 diagnosed with LBP at follow-ups) were included in these quantitative analyses. These studies included individuals who were pain-free at baseline or who never had prior LBP to reflect the true incidences/risk factors that can help inform future primary prevention strategies.

In the present review, most of the included studies were of fair-quality. Commonly observed reporting weaknesses included failure to provide information regarding sample size justification, power description or effect estimate. Further weaknesses included failure to assess exposures more than once over time, failure to blind assessors, and losing more than 20% of participants at follow-up. Furthermore, the adjustments for confounding factors varied among studies, whereas some studies did not adjust for important factors that may confound the association between sedentary behavior and LBP such as physical activity level, psychosocial variables, smoking, body mass index and occupational risk factors. Future studies are recommended to adjust for these important factors which have been shown to be associated with LBP (Alzahrani et al., 2019; Burström, Nilsson & Wahlström, 2015; Shiri et al., 2010; Taylor et al., 2014; Zhang et al., 2018).

In terms of measurements used in assessing sedentary behavior, all the studies except one used self-reported measures. Self-reported measures have been shown to be prone to underestimating sedentary behavior and recall bias. Future research could consider using objective measures such as accelerometer-based devices to accurately detect time spent in sitting time (Atkin et al., 2012). However, unlike context-specific self-reports, they do not provide contextual information on patterns of sedentary behavior. For example, in the case of sitting, it should be examined in combination with whole-body vibrations or awkward postures which have been identified as risk factors for LBP (Burström, Nilsson & Wahlström, 2015; Lis et al., 2007). Therefore, future studies are recommended to use a combination of both self-reported and accelerometer-based measures in assessing sedentary behaviors.

The results were changed when we used different methods of categorizing sedentary time by using unbounded open categories (>2, >4, >6 and >8 h/d). The findings of the sensitivity analyses showed that the sedentary duration exceeding 2, 4 or 6 h/d was associated with a lower risk of LBP. However, this association disappeared when the analysis was restricted to sedentary duration exceeding 8 h/d. Given that the ORs for >2, >4, >6 and >8 h/d were basically the same, the fact that the OR for >8 h/d is not quite statistically significant is unimportant. It should be noted that the magnitudes of ORs did not vary significantly among all categories of sedentary duration where the OR was 0.94 for the sedentary durations of >2, >4, or >6 h/d and it was 0.92 for the sedentary duration of >8 h/d. Furthermore, the magnitudes of the ORs were very close to 1.00 which might imply trend toward no association between sedentary behavior and LBP. When the studies were considered individually, we found that all included studies, except two studies, did not show significant associations between sedentary behavior and the risk of LBP. One of these studies was by Macfarlane et al. (1997) that showed that sedentary behavior exceeding 2 h/d was associated with a lower risk of LBP in female participants. The other study was by Venseth (2014) that found that sedentary behavior of 9–10 h/d was linked to a lower risk of LBP. Nevertheless, the differing methods used in previous studies related to categorizing the sedentary duration may explain some of the inconsistency among studies.

The main strengths of this review are (1) conducting a comprehensive literature search strategy including five databases; (2) conducting meta-analyses of longitudinal studies (with an average follow-up period of 6.29 years) to determine the association between sedentary behavior and LBP; and (3) included fully adjusted models from each study in the analyses to account for potential confounding factors. Several limitations should be considered when interpreting the results of this review. First, the number of the included studies in the meta-analyses was small. Second, all included studies except one used self-reported measures of sedentary behavior, which may induce recall bias and false estimation. Future research investigating sedentary behavior in people with LBP could consider also using an objective measure, to accurately detect time spent in sedentary behavior (Atkin et al., 2012). Third, measurements and classification of sedentary behavior in terms of duration differed across studies, which may induce the misclassification of sedentary behavior amounts. Fourth, the sensitivity analyses could not be conducted since only two of the studies included in the quantitative syntheses were classified as good (high quality). Fifth, all the included studies were from high-income countries, which may not be generalized to middle- and low-income countries.

## Conclusions

This review found that sedentary behavior of different durations (3–<6, 6–8 or >8 h/d) was not associated with LBP. Furthermore, our results showed that sedentary behavior for ≥3 h/d can lead to worse LBP-related disability. However, these conclusions are tentative as the evidence was derived from mostly fair-quality studies using subjective measures of sedentary behavior.

## Supplemental Information

10.7717/peerj.13127/supp-1 Supplemental Information 1 Supplementary Figures and Tables.

10.7717/peerj.13127/supp-2 Supplemental Information 2 PRISMA checklist.

10.7717/peerj.13127/supp-3 Supplemental Information 3 Contribution and Rationale.

## Additional Information and Declarations

### Data Availability

The following information was supplied regarding data availability: This systematic review reviewed: Medline *via* OvidSP (1946–present), CINAHL *via* EBSCOhost (1981–present), Scopus, EMBASE (1947–present) and Web of Science.

## References

[ref-1] Alzahrani H, Mackey M, Stamatakis E, Zadro JR, Shirley D (2019). The association between physical activity and low back pain: a systematic review and meta-analysis of observational studies. Scientific Reports.

[ref-2] Amorim AB, Levy GM, Pérez-Riquelme F, Simic M, Pappas E, Dario AB, Ferreira ML, Carrillo E, Luque-Suarez A, Ordoñana JR, Ferreira PH (2017). Does sedentary behavior increase the risk of low back pain? A population-based co-twin study of Spanish twins. Spine Journal.

[ref-3] Andersen JH, Haahr JP, Frost P (2007). Risk factors for more severe regional musculoskeletal symptoms: a two-year prospective study of a general working population. Arthritis and Rheumatism.

[ref-4] Atkin AJ, Gorely T, Clemes SA, Yates T, Edwardson C, Brage S, Salmon J, Marshall SJ, Biddle SJ (2012). Methods of measurement in epidemiology: sedentary behaviour. International Journal of Epidemiology.

[ref-5] Balling M, Holmberg T, Petersen CB, Aadahl M, Meyrowitsch DW, Tolstrup JS (2019). Total sitting time, leisure time physical activity and risk of hospitalization due to low back pain: the danish health examination survey cohort 2007–2008. Scandinavian Journal of Public Health.

[ref-7] Barnes J, Behrens TK, Benden ME, Biddle S, Bond D, Brassard P, Brown H, Carr L, Chaput J-P, Christian H (2012). Letter to the editor: standardized use of the terms “sedentary” and “sedentary behaviours”. Applied Physiology, Nutrition, and Metabolism.

[ref-8] Beach TAC, Parkinson RJ, Stothart JP, Callaghan JP (2005). Effects of prolonged sitting on the passive flexion stiffness of the in vivo lumbar spine. Spine Journal.

[ref-9] Behrens G, Leitzmann MF (2013). The association between physical activity and renal cancer: systematic review and meta-analysis. British Journal of Cancer.

[ref-10] Billy GG, Lemieux SK, Chow MX (2014). Changes in lumbar disk morphology associated with prolonged sitting assessed by magnetic resonance imaging. PM&R.

[ref-11] Blough J, Loprinzi PD (2018). Experimentally investigating the joint effects of physical activity and sedentary behavior on depression and anxiety: a randomized controlled trial. Journal of Affective Disorders.

[ref-12] Burström L, Nilsson T, Wahlström J (2015). Whole-body vibration and the risk of low back pain and sciatica: a systematic review and meta-analysis. International Archives of Occupational and Environmental Health.

[ref-13] Chen SM, Liu MF, Cook J, Bass S, Lo SK (2009). Sedentary lifestyle as a risk factor for low back pain: a systematic review. International Archives of Occupational and Environmental Health.

[ref-14] Clair C, Cohen MJ, Eichler F, Selby KJ, Rigotti NA (2015). The effect of cigarette smoking on diabetic peripheral neuropathy: a systematic review and meta-analysis. Journal of General Internal Medicine.

[ref-15] Dumuid D, Pedišić Ž, Palarea-Albaladejo J, Martín-Fernández JA, Hron K, Olds T (2020). Compositional data analysis in time-use epidemiology: what, why, how. International Journal of Environmental Research and Public Health.

[ref-16] Edwardson CL, Gorely T, Davies MJ, Gray LJ, Khunti K, Wilmot EG, Yates T, Biddle SJ (2012). Association of sedentary behaviour with metabolic syndrome: a meta-analysis. PLOS ONE.

[ref-17] Egger M, Davey Smith G, Schneider M, Minder C (1997). Bias in meta-analysis detected by a simple, graphical test. BMJ.

[ref-18] Gupta N, Christiansen CS, Hallman DM, Korshoj M, Carneiro IG, Holtermann A (2015). Is objectively measured sitting time associated with low back pain? A cross-sectional investigation in the NOMAD study. PLOS ONE.

[ref-19] Hamer M, Coombs N, Stamatakis E (2014). Associations between objectively assessed and self-reported sedentary time with mental health in adults: an analysis of data from the Health Survey for England. BMJ Open.

[ref-20] Harkness EF, Macfarlane GJ, Nahit ES, Silman AJ, McBeth J (2003). Risk factors for new-onset low back pain amongst cohorts of newly employed workers. Rheumatology.

[ref-21] Hartvigsen J, Bakketeig LS, Leboeuf-Yde C, Engberg M, Lauritzen T (2001). The association between physical workload and low back pain clouded by the “healthy worker” effect: population-based cross-sectional and 5-year prospective questionnaire study. Spine.

[ref-22] Hartvigsen J, Christensen K (2007). Active lifestyle protects against incident low back pain in seniors: a population-based 2-year prospective study of 1387 Danish twins aged 70–100 years. Spine.

[ref-23] Hartvigsen J, Leboeuf-Yde C, Lings S, Corder EH (2000). Is sitting-while-at-work associated with low back pain? A systematic, critical literature review. Scandinavian Journal of Public Health.

[ref-24] Hestbaek L, Larsen K, Weidick F, Leboeuf-Yde C (2005). Low back pain in military recruits in relation to social background and previous low back pain. A cross-sectional and prospective observational survey. BMC Musculoskeletal Disorders.

[ref-25] Hicks CM (2009). Research methods for clinical therapists: applied project design and analysis.

[ref-26] Higgins JP, Green S (2011). Cochrane handbook for systematic reviews of interventions.

[ref-27] Higgins JP, Thompson SG (2002). Quantifying heterogeneity in a meta-analysis. Statistics in Medicine.

[ref-28] Higgins JP, Thompson SG, Deeks JJ, Altman DG (2003). Measuring inconsistency in meta-analyses. BMJ.

[ref-29] Hoy D, Bain C, Williams G, March L, Brooks P, Blyth F, Woolf A, Vos T, Buchbinder R (2012). A systematic review of the global prevalence of low back pain. Arthritis and Rheumatism.

[ref-30] Hussain SM, Urquhart DM, Wang Y, Dunstan D, Shaw JE, Magliano DJ, Wluka AE, Cicuttini FM (2016). Associations between television viewing and physical activity and low back pain in community-based adults: a cohort study. Medicine.

[ref-31] Jensen C, Ryholt C, Burr H, Villadsen E, Christensen HJW (2002). Work-related psychosocial, physical and individual factors associated with musculoskeletal symptoms in computer users. Work and Stress.

[ref-32] Juul-Kristensen B, Sogaard K, Stroyer J, Jensen C (2004). Computer users’ risk factors for developing shoulder, elbow and back symptoms. Scandinavian Journal of Work, Environment & Health.

[ref-33] Katzmarzyk PT, Church TS, Craig CL, Bouchard C (2009). Sitting time and mortality from all causes, cardiovascular disease, and cancer. Medicine & Science in Sports & Exercise.

[ref-34] Kodama S, Tanaka S, Heianza Y, Fujihara K, Horikawa C, Shimano H, Saito K, Yamada N, Ohashi Y, Sone H (2013). Association between physical activity and risk of all-cause mortality and cardiovascular disease in patients with diabetes. A meta-analysis. Diabetes Care.

[ref-35] Kong PW, Lim CT, Goh JCH (2010). Changes in perceived comfort, strength and electromyographic response in lower back, hip and leg muscles during 8-hour prolonged sitting. 6th World Congress of Biomechanics (WCB 2010) August 1–6, 2010 Singapore: In Conjunction with 14th International Conference on Biomedical Engineering (ICBME) and 5th Asia Pacific Conference on Biomechanics (APBiomech).

[ref-36] Kopec JA, Sayre EC, Esdaile JM (2004). Predictors of back pain in a general population cohort. Spine.

[ref-37] Liao Y, Shibata A, Ishii K, Oka K (2016). Independent and combined associations of physical activity and sedentary behavior with depressive symptoms among japanese adults. International Journal of Behavioral Medicine.

[ref-38] Lis AM, Black KM, Korn H, Nordin M (2007). Association between sitting and occupational LBP. European Spine Journal.

[ref-39] Lunde LK, Koch M, Knardahl S, Veiersted KB (2017). Associations of objectively measured sitting and standing with low-back pain intensity: a 6-month follow-up of construction and healthcare workers. Scandinavian Journal of Work, Environment & Health.

[ref-40] Macfarlane GJ, Thomas E, Papageorgiou AC, Croft PR, Jayson MI, Silman AJ (1997). Employment and physical work activities as predictors of future low back pain. Spine.

[ref-41] Matsudaira K, Konishi H, Miyoshi K, Isomura T, Takeshita K, Hara N, Yamada K, Machida H (2012). Potential risk factors for new onset of back pain disability in Japanese workers: findings from the Japan epidemiological research of occupation-related back pain study. Spine.

[ref-42] Matthews CE, Chen KY, Freedson PS, Buchowski MS, Beech BM, Pate RR, Troiano RP (2008). Amount of time spent in sedentary behaviors in the United States, 2003–2004. American Journal of Epidemiology.

[ref-43] Moher D, Liberati A, Tetzlaff J, Altman DG, The PRISMA Group (2009). Preferred reporting items for systematic reviews and meta-analyses: the PRISMA statement. PLOS Medicine.

[ref-44] National Institutes of Health (2014). Quality assessment tool for observational cohort and cross-sectional studies. https://www.nhlbi.nih.gov/health-pro/guidelines/in-develop/cardiovascular-risk-reduction/tools/cohort.

[ref-46] Omokhodion FO, Sanya AO (2003). Risk factors for low back pain among office workers in Ibadan, Southwest Nigeria. Occupational Medicine.

[ref-47] Owen N, Sparling PB, Healy GN, Dunstan DW, Matthews CE (2010). Sedentary behavior: emerging evidence for a new health risk.

[ref-48] Pinheiro MB, Ferreira ML, Refshauge K, Maher CG, Ordoñana JR, Andrade TB, Tsathas A, Ferreira PH (2016). Symptoms of depression as a prognostic factor for low back pain: a systematic review. Spine Journal.

[ref-49] RevMan The Cochrane Collaboration (2014). Review manager (RevMan). 5.3.

[ref-50] San Giorgi MR, Helder HM, Lindeman RJS, de Bock GH, Dikkers FG (2016). The association between gastroesophageal reflux disease and recurrent respiratory papillomatosis: a systematic review. Laryngoscope.

[ref-51] Shanahan MJ, Flaherty BP (2001). Dynamic patterns of time use in adolescence. Child Development.

[ref-52] Shiri R, Falah-Hassani K, Heliovaara M, Solovieva S, Amiri S, Lallukka T, Burdorf A, Husgafvel-Pursiainen K, Viikari-Juntura E (2019). Risk factors for low back pain: a population-based longitudinal study. Arthritis Care & Research.

[ref-53] Shiri R, Karppinen J, Leino-Arjas P, Solovieva S, Viikari-Juntura E (2010). The association between smoking and low back pain: a meta-analysis. The American Journal of Medicine.

[ref-54] Sterne JAC, Sutton AJ, Ioannidis JPA, Terrin N, Jones DR, Lau J, Carpenter J, Rucker G, Harbord RM, Schmid CH, Tetzlaff J, Deeks JJ, Peters J, Macaskill P, Schwarzer G, Duval S, Altman DG, Moher D, Higgins JPT (2011). Recommendations for examining and interpreting funnel plot asymmetry in meta-analyses of randomised controlled trials. BMJ.

[ref-55] Swinkels-Meewisse IE, Roelofs J, Verbeek AL, Oostendorp RA, Vlaeyen JW (2003). Fear of movement/(re)injury, disability and participation in acute low back pain. Pain.

[ref-56] Tacconelli E (2010). Systematic reviews: CRD’s guidance for undertaking reviews in health care. Lancet Infectious Diseases.

[ref-57] Taylor JB, Goode AP, George SZ, Cook CE (2014). Incidence and risk factors for first-time incident low back pain: a systematic review and meta-analysis. The Spine Journal.

[ref-58] Teychenne M, Costigan SA, Parker K (2015). The association between sedentary behaviour and risk of anxiety: a systematic review. BMC Public Health.

[ref-59] Thorp AA, Owen N, Neuhaus M, Dunstan DW (2011). Sedentary behaviors and subsequent health outcomes in adults a systematic review of longitudinal studies, 1996–2011. American Journal of Preventive Medicine.

[ref-60] Valat JP (2005). Factors involved in progression to chronicity of mechanical low back pain. Joint Bone Spine.

[ref-61] van Tulder M, Koes B, Bombardier C (2002). Low back pain. Best Practice & Research Clinical Rheumatology.

[ref-62] van Tulder M, Becker A, Bekkering T, Breen A, del Real MT, Hutchinson A, Koes B, Laerum E, Malmivaara A, COST B13 Working Group on Guidelines for the Management of Acute Low Back Pain in Primary Care (2006). Chapter 3. European guidelines for the management of acute nonspecific low back pain in primary care. European Spine Journal.

[ref-63] Venseth TB (2014). Physical activity and time spent sitting as a risk factor for low-back pain: longitudinal data from the HUNT studyMaster thesis.

[ref-64] Vlaeyen JW, Kole-Snijders AM, Rotteveel AM, Ruesink R, Heuts PH (1995). The role of fear of movement/(re)injury in pain disability. Journal of Occupational Rehabilitation.

[ref-65] Walker BF (2000). The prevalence of low back pain: a systematic review of the literature from 1966 to 1998. Journal of Spinal Disorders.

[ref-66] Wu A, March L, Zheng X, Huang J, Wang X, Zhao J, Blyth FM, Smith E, Buchbinder R, Hoy D (2020). Global low back pain prevalence and years lived with disability from 1990 to 2017: estimates from the Global Burden of Disease Study 2017. Annals of Translational Medicine.

[ref-67] Xu Y, Bach E, Orhede E (1997). Work environment and low back pain: the influence of occupational activities. Occupational and Environmental Medicine.

[ref-68] Yip VYB (2004). New low back pain in nurses: work activities, work stress and sedentary lifestyle. Journal of Advanced Nursing.

[ref-69] Zhang T-T, Liu Z, Liu Y-L, Zhao J-J, Liu D-W, Tian Q-B (2018). Obesity as a risk factor for low back pain: a meta-analysis. Clinical Spine Surgery: A Spine Publication.

