# Supplementary material for: The association between sedentary behavior and low back pain in adults: a systematic review and meta-analysis of longitudinal studies
Source: PeerJ. 2022 Mar 28;10:e13127. doi: 10.7717/peerj.13127 (PMC8983064; doi:10.7717/peerj.13127)
Supplement: Supplemental Information 1 [file peerj-10-13127-s001.docx]

**Supplementary File**

**The Association between Sedentary Behavior and Low Back Pain in Adults: A Systematic Review and Meta-Analysis of Longitudinal Studies**

**Supplementary Tables and Figures**

**List (In order of appearance in manuscript text):**

**Supplementary Table 1.** Search strategy (Medline).

**Supplementary Table 2.** National Institutes of Health’s Quality Assessment Tool to assess sedentary behaviour in low back pain populations.

**Supplementary Table 3.** Studies included in the meta-analyses.

**Supplementary Table 4.** Methodological quality assessment of included studies.

**Supplementary Table 5.** Comparison groups included in the meta-analyses.

**Supplementary Figure 1.** Association between sedentary behavior > 2 hours vs. lowest and low back pain.

**Supplementary Figure 2.** Association between sedentary behavior > 4 hours vs. lowest and low back pain.

**Supplementary Figure 3.** Association between sedentary behavior > 6 hours vs. lowest and low back pain.

**Supplementary Figure 4.** Association between sedentary behavior > 8 hours vs. lowest and low back pain.

**Supplementary Table 1. Search strategy (Medline)**

| **Search** | **Query** |
| --- | --- |
| 1 | Sedentar*.tw. |
| 2 | Lifestyle.tw. |
| 3 | occupation*.tw. |
| 4 | work*.tw. |
| 5 | sit*.tw. |
| 6 | seat*.tw. |
| 7 | "office work*".tw. |
| 8 | "white-collar work*".tw. |
| 9 | "computer work*".tw. |
| 10 | (television adj3 watch*).tw. |
| 11 | (TV adj3 watch*).tw. |
| 12 | "computer gam*".tw. |
| 13 | "video gam*".tw. |
| 14 | internet.tw. |
| 15 | exp Back Pain/ |
| 16 | exp Low Back Pain/ |
| 17 | "back pain".mp. |
| 18 | "backpain".mp. |
| 19 | "back ache".mp. |
| 20 | "backache$".mp. |
| 21 | "spinal pain".mp. |
| 22 | dorsalgia$.tw,kf. |
| 23 | lumbago.tw,kf. |
| 24 | back disorder$.tw,kf. |
| 25 | (lumb$ adj3 pain).tw,kf. |
| 26 | "vertebral pain".mp. |
| 27 | 15 or 16 or 17 or 18 or 19 or 20 or 21 or 22 or 23 or 24 or 25 or 26 |
| 28 | 1 or 2 or 3 or 4 or 5 or 6 or 7 or 8 or 9 or 10 or 11 or 12 or 13 or 14 |
| 29 | 27 and 28 |

**Supplementary Table 2.** **National Institutes of Health’s Quality Assessment Tool to assess sedentary behaviour in low back pain populations**

| Criteria | Yes | No | Other  (CD, NR, NA)* |
| --- | --- | --- | --- |
| 1. Was the research question or objective in this paper clearly stated? |  |  |  |
| 2. Was the study population clearly specified and defined? |  |  |  |
| 3. Was the participation rate of eligible persons at least 50%? |  |  |  |
| 4. Were all the subjects selected or recruited from the same or similar populations (including the same time period)? Were inclusion and exclusion criteria for being in the study prespecified and applied uniformly to all participants? |  |  |  |
| 5. Was a sample size justification, power description, or variance and effect estimates provided? |  |  |  |
| 6. For the analyses in this paper, were the exposure(s) of interest measured prior to the outcome(s) being measured? |  |  |  |
| 7. Was the timeframe sufficient so that one could reasonably expect to see an association between exposure and outcome if it existed? |  |  |  |
| 8. For exposures that can vary in amount or level, did the study examine different levels of the exposure as related to the outcome (e.g., categories of exposure, or exposure measured as continuous variable)? |  |  |  |
| 9. Were the exposure measures (independent variables) clearly defined, valid, reliable, and implemented consistently across all study participants? |  |  |  |
| 10. Was the exposure(s) assessed more than once over time? |  |  |  |
| 11. Were the outcome measures (dependent variables) clearly defined, valid, reliable, and implemented consistently across all study participants? |  |  |  |
| 12. Were the outcome assessors blinded to the exposure status of participants? |  |  |  |
| 13. Was loss to follow-up after baseline 20% or less? |  |  |  |
| 14. Were key potential confounding variables measured and adjusted statistically for their impact on the relationship between exposure(s) and outcome(s)? |  |  |  |

*CD, cannot determine; NA, not applicable; NR, not reported.

| **Quality Rating (Good, Fair, or Poor)** |
| --- |
| Rater #1 initials: |
| Rater #2 initials: |
| Additional Comments (If POOR, please state why): |

For more information on the tool: https://www.nhlbi.nih.gov/health-topics/study-quality-assessment-tools

**Supplementary Table 3. Studies included in the meta-analyses**

| **First author (year)** | **Exposure (sedentary behaviour measured)** | **Outcome** | **Follow-up time** | **Adjusted confounding factors** |
| --- | --- | --- | --- | --- |
| Balling,Holmberg,Petersen et al. (2019) | Sitting (h/d) | Prevalence of LBP | 7.4 years | Age, gender, mental disorder, education, smoking status, BMI, leisure time physical activity and physical activity at work |
| Harkness,Macfarlane,Nahit et al. (2003) | Sitting (h/d) | New onset LBP lasting for at least 24 hours in the past month | 2 years | Age, gender, occupation, standing, kneeling, squatting, bending, stretching below knee level, and working with hands above shoulder |
| Hussain,Urquhart,Wang et al. (2016) | Television viewing (h/d) | LBP intensity and disability | 15 years | Age, gender (stratified), education, smoking status, dietary guideline index score, BMI, mental component score of SF-36, and physical activity |
| Juul-Kristensen,Sogaard,Stroyer et al. (2004) | Working on computer (percentage of time) | Frequency and intensity of LBP in the last 12 months | 1.9 years | Age, gender, work-factor, ergonomic, and psychosocial variables |
| Macfarlane,Thomas,Papageorgiou et al. (1997) | Sitting (h/d) | Episodes of LBP occurring in the last 12 months | 1 year | Age and gender |
| Matsudaira,Konishi,Miyoshi et al. (2012) | Desk work (h/d) | New onset LBP in the last 24 months | 2 years | Age, gender, and previous episodes of LBP |
| Shiri,Falah-Hassani,Heliovaara et al. (2019) | Sitting (h/d) | LBP for >7 days in the last 12 months | 11 years | Age and gender |
| Venseth (2014) | Sitting (h/d) | New onset of LBP lasting for 3 consecutive months in the last 12 months | 10 years | Age, gender, BMI, smoking, education and psychosocial well being |
| BMI, body mass index; LBP, low back pain. | | | | |

**Supplementary Table 4. Methodological quality assessment of included longitudinal studies**

| **First author (year)** | **1** | **2** | **3** | **4** | **5** | **6** | **7** | **8** | **9** | **10** | **11** | **12** | **13** | **14** | **Total score (14)** | **Quality of Study*** | |
| --- | --- | --- | --- | --- | --- | --- | --- | --- | --- | --- | --- | --- | --- | --- | --- | --- | --- |
| Amorim,Levy,Perez-Riquelme et al. (2017) | Y | Y | Y | N | Y | Y | NA | CD | CD | N | CD | Y | Y | Y | 8 | 57.1 | Fair |
| Andersen,Haahr & Frost (2007) | Y | Y | Y | CD | NR | Y | Y | Y | Y | Y | Y | NR | Y | Y | 11 | 78.6 | Good |
| Balling,Holmberg,Petersen et al. (2019) | Y | Y | N | Y | NR | Y | Y | Y | Y | N | Y | NR | N | Y | 9 | 64.3 | Fair |
| Harkness,Macfarlane,Nahit et al. (2003) | Y | Y | Y | Y | Y | Y | Y | Y | Y | Y | Y | NR | N | Y | 12 | 85.7 | Good |
| Hartvigsen & Christensen (2007) | Y | Y | NR | Y | NR | Y | Y | Y | Y | CD | Y | NR | N | Y | 9 | 64.3 | Fair |
| Hartvigsen,Bakketeig,Leboeuf-Yde et al. (2001) | Y | Y | Y | N | Y | CD | Y | Y | CD | Y | Y | NR | Y | Y | 10 | 71.4 | Fair |
| Hestbaek,Larsen,Weidick et al. (2005) | Y | Y | CD | Y | NR | Y | CD | Y | Y | Y | Y | NR | N | Y | 9 | 64.3 | Fair |
| Hussain,Urquhart,Wang et al. (2016) | Y | Y | NR | Y | NR | Y | NR | Y | Y | Y | Y | NR | N | Y | 9 | 64.3 | Fair |
| Juul-Kristensen,Sogaard,Stroyer et al. (2004) | Y | Y | Y | Y | N | Y | Y | Y | CD | Y | Y | N | Y | Y | 11 | 78.6 | Good |
| Kopec,Sayre & Esdaile (2004) | Y | Y | Y | Y | N | Y | Y | Y | CD | Y | Y | N | Y | Y | 11 | 78.6 | Good |
| Lunde,Koch,Knardahl et al. (2017) | Y | Y | N | Y | N | Y | N | Y | CD | Y | Y | N | N | Y | 8 | 57.1 | Fair |
| Macfarlane,Thomas,Papageorgiou et al. (1997) | Y | Y | Y | Y | N | Y | Y | Y | N | N | Y | NR | N | N | 8 | 57.1 | Fair |
| Matsudaira,Konishi,Miyoshi et al. (2012) | Y | Y | Y | Y | N | Y | Y | Y | CD | Y | Y | Y | Y | N | 11 | 78.6 | Good |
| Shiri,Falah-Hassani,Heliovaara et al. (2019) | Y | Y | Y | Y | NR | Y | Y | Y | CD | N | Y | NR | N | N | 8 | 57.1 | Fair |
| Venseth (2014) | Y | Y | Y | Y | NR | Y | Y | Y | Y | N | N | NR | Y | Y | 10 | 71.4 | Fair |
| Yip (2004) | Y | Y | NR | Y | Y | Y | Y | Y | Y | N | Y | NR | N | N | 9 | 64.3 | Fair |
| Y, yes; N, no; CD, cannot determine; NA, not applicable; NR, not reported. | | | | | | | | | | | | | | | | | |
| * Divide the total score by 14 and multiply by 100 ((Total score/14)*100).  Quality of study: Good (75-100%), Fair (25-75%), or poor (0-25%). | | | | | | | | | | | | | | | | | |

| 1. Association between sedentary behavior (6 – 8 h/d) and prevalence of low back pain | | | | |
| --- | --- | --- | --- | --- |
|  | Sedentary behaviour | | | |
|  | Type | Duration (h/d) | Estimated time (h/d) | Reference category (h/d) |
| Balling,Holmberg,Petersen et al. (2019) | Time spent sitting | 6 to < 10 | 8 | < 6 |
| Juul-Kristensen,Sogaard,Stroyer et al. (2004) (a) | Working on computer | 75% of time | 6 | < 25% of time (< 2) |
| Juul-Kristensen,Sogaard,Stroyer et al. (2004) (b) | Working on computer | All the time with computer | 8 | < 25% of time (< 2) |
| Shiri,Falah-Hassani,Heliovaara et al. (2019) | Time spent sitting | ≥ 5 | 7.5 | < 5 |
| Venseth (2014) | Time spent sitting | 7–8 | 7.5 | < 5 |

**Supplementary Table 5. Comparison groups included in the meta-analyses**

| 1. Association between sedentary behavior (3 – < 6 h/d) and prevalence of low back pain | | | | |
| --- | --- | --- | --- | --- |
|  | Sedentary behaviour | | | |
|  | Type | Duration (h/d) | Estimated time (h/d) | Reference category (h/d) |
| Harkness,Macfarlane,Nahit et al. (2003) | Sitting at work | ≥ 2 | 3 | Do not sit as part of job (0) |
| Juul-Kristensen,Sogaard,Stroyer et al. (2004) | Working on computer | 50% of time | 4 | < 25% of time (< 2) |
| Macfarlane,Thomas,Papageorgiou et al. (1997) (Female) | Sitting as part of job | > 2 | 3 | < 2 |
| Macfarlane,Thomas,Papageorgiou et al. (1997) (Male) | Sitting as part of job | > 2 | 3 | < 2 |
| Venseth (2014) | Time spent sitting | 5–6 | 5.5 | < 5 |

| 1. Association between sedentary behavior (> 8 h/d) and prevalence of low back pain | | | | |
| --- | --- | --- | --- | --- |
|  | Sedentary behaviour | | | |
|  | Type | Duration (h/d) | Estimated duration (h/d) | Reference category (h/d) |
| Balling,Holmberg,Petersen et al. (2019) | Time spent sitting | ≥ 10 | 12 | < 6 |
| Matsudaira,Konishi,Miyoshi et al. (2012) | Desk work | ≥ 6 | 9 | < 6 |
| Venseth (2014) (a) | Time spent sitting | 9–10 | 9.5 | < 5 |
| Venseth (2014) (b) | Time spent sitting | >11 | 11.5 | < 5 |

| 1. Association between sedentary behavior (≥ 3 h/d) and low back pain-related outcomes | | | | |
| --- | --- | --- | --- | --- |
|  | Sedentary behaviour | | | |
|  | Type | Duration (h/d) | Estimated duration (h/d) | Reference category |
| - Pain intensity | | | | |
| Hussain,Urquhart,Wang et al. (2016) (Female) | Television viewing | ≥ 2 | 3 | < 2 |
| Hussain,Urquhart,Wang et al. (2016) (Male) | Television viewing | ≥ 2 | 3 | < 2 |
| Juul-Kristensen,Sogaard,Stroyer et al. (2004) (a) | Working on computer | 50% of time | 4 | < 25% of time (< 2) |
| Juul-Kristensen,Sogaard,Stroyer et al. (2004) (b) | Working on computer | 75% of time | 6 | < 25% of time (< 2) |
| Juul-Kristensen,Sogaard,Stroyer et al. (2004) (c) | Working on computer | All time with computer | 8 | < 25% of time (< 2) |
|  | | | | |
| - LBP-related disability | | | | |
| Hussain,Urquhart,Wang et al. (2016) (Female) | Television viewing | ≥ 2 | 3 | < 2 |
| Hussain,Urquhart,Wang et al. (2016) (Male) | Television viewing | ≥ 2 | 3 | < 2 |


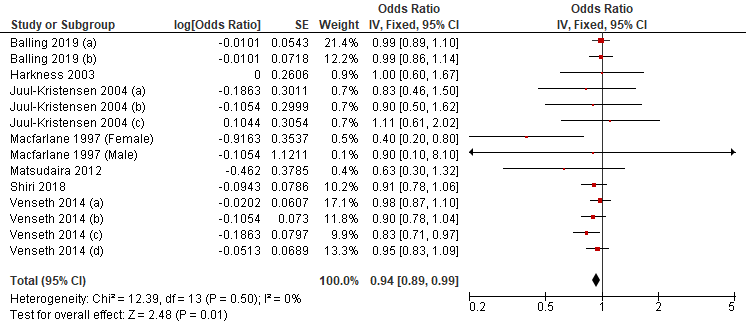


**Supplementary Figure 1. Association between sedentary behavior > 2 hours vs. lowest and low back pain.**


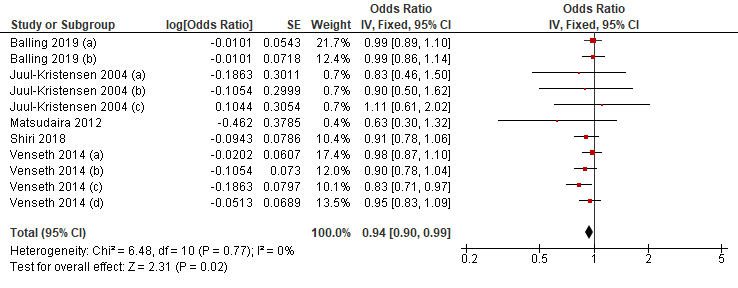


**Supplementary Figure 2. Association between sedentary behavior > 4 hours vs. lowest and low back pain.**


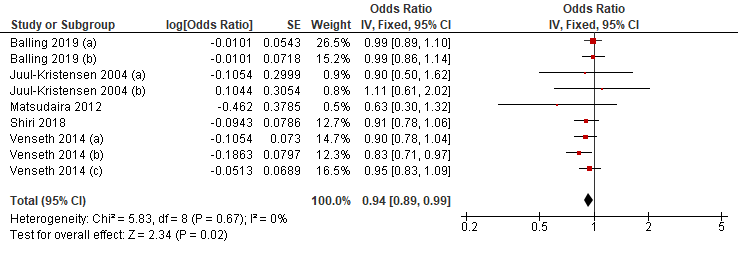


**Supplementary Figure 3. Association between sedentary behavior > 6 hours vs. lowest and low back pain.**


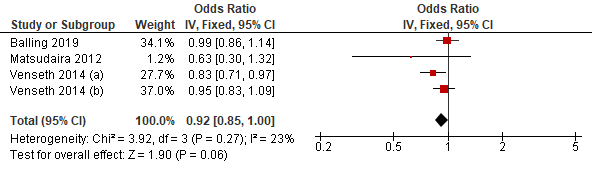


**Supplementary Figure 4. Association between sedentary behavior > 8 hours vs. lowest and low back pain.**

**References**

Amorim AB, Levy GM, Perez-Riquelme F, Simic M, Pappas E, Dario AB, Ferreira ML, Carrillo E, Luque-Suarez A, Ordonana JR et al. . 2017. Does sedentary behavior increase the risk of low back pain? A population-based co-twin study of Spanish twins. *Spine Journal: Official Journal of the North American Spine Society* 17:933-942.

Andersen JH, Haahr JP, and Frost P. 2007. Risk factors for more severe regional musculoskeletal symptoms: a two-year prospective study of a general working population. *Arthritis & Rheumatism* 56:1355-1364.

Balling M, Holmberg T, Petersen CB, Aadahl M, Meyrowitsch DW, and Tolstrup JS. 2019. Total sitting time, leisure time physical activity and risk of hospitalization due to low back pain: The Danish Health Examination Survey cohort 2007-2008. *Scand J Public Health* 47:45-52. 10.1177/1403494818758843

Harkness EF, Macfarlane GJ, Nahit ES, Silman AJ, and McBeth J. 2003. Risk factors for new-onset low back pain amongst cohorts of newly employed workers. *Rheumatology* 42:959-968. 10.1093/rheumatology/keg265

Hartvigsen J, Bakketeig LS, Leboeuf-Yde C, Engberg M, and Lauritzen T. 2001. The association between physical workload and low back pain clouded by the "healthy worker" effect: population-based cross-sectional and 5-year prospective questionnaire study. *Spine* 26:1788-1792; discussion 1792-1783.

Hartvigsen J, and Christensen K. 2007. Active lifestyle protects against incident low back pain in seniors: a population-based 2-year prospective study of 1387 Danish twins aged 70-100 years. *Spine (03622436)* 32:76-81.

Hestbaek L, Larsen K, Weidick F, and Leboeuf-Yde C. 2005. Low back pain in military recruits in relation to social background and previous low back pain. A cross-sectional and prospective observational survey. *BMC Musculoskeletal Disorders* 6:25.

Hussain SM, Urquhart DM, Wang Y, Dunstan D, Shaw JE, Magliano DJ, Wluka AE, and Cicuttini FM. 2016. Associations between television viewing and physical activity and low back pain in community-based adults: A cohort study. *Medicine* 95:e3963.

Juul-Kristensen B, Sogaard K, Stroyer J, and Jensen C. 2004. Computer users' risk factors for developing shoulder, elbow and back symptoms. *Scandinavian Journal of Work, Environment & Health* 30:390-398.

Kopec JA, Sayre EC, and Esdaile JM. 2004. Predictors of back pain in a general population cohort. *Spine* 29:70-77; discussion 77-78.

Lunde LK, Koch M, Knardahl S, and Veiersted KB. 2017. Associations of objectively measured sitting and standing with low-back pain intensity: a 6-month follow-up of construction and healthcare workers. *Scandinavian Journal of Work, Environment & Health* 43:269-278.

Macfarlane GJ, Thomas E, Papageorgiou AC, Croft PR, Jayson MI, and Silman AJ. 1997. Employment and physical work activities as predictors of future low back pain. *Spine* 22:1143-1149.

Matsudaira K, Konishi H, Miyoshi K, Isomura T, Takeshita K, Hara N, Yamada K, and Machida H. 2012. Potential risk factors for new onset of back pain disability in Japanese workers: findings from the Japan epidemiological research of occupation-related back pain study. *Spine* 37:1324-1333.

Shiri R, Falah-Hassani K, Heliovaara M, Solovieva S, Amiri S, Lallukka T, Burdorf A, Husgafvel-Pursiainen K, and Viikari-Juntura E. 2019. Risk Factors for Low Back Pain: A Population-Based Longitudinal Study. *Arthritis Care Res (Hoboken)* 71:290-299. 10.1002/acr.23710

Venseth TB. 2014. Physical activity and time spent sitting as a risk factor for low-back pain: longitudinal data from the HUNT studyMaster thesis. Norwegian University of Science and Technology.

Yip VYB. 2004. New low back pain in nurses: work activities, work stress and sedentary lifestyle. *Journal of Advanced Nursing* 46:430-440. 10.1111/j.1365-2648.2004.03009.x
