# Supplementary material for: The association between sedentary behavior and low back pain in adults: a systematic review and meta-analysis of longitudinal studies
Source: PeerJ. 2022 Mar 28;10:e13127. doi: 10.7717/peerj.13127 (PMC8983064; doi:10.7717/peerj.13127)
Supplement: Supplemental Information 3 [file peerj-10-13127-s003.docx]

1. **The rationale for conducting the systematic review**

Despite spending a longer time in sedentary behaviors is linked to several health problems, the quantitative risk of low back pain (LBP) associated with different amounts of sedentary behavior is still unknown. Hence, no consensus recommendations have been issued regarding limits on the amount of sedentary time to optimize LBP prevention. To address this knowledge gap, we performed a pooled analysis of longitudinal studies using a meta-analytical approach to examine the association between sedentary time and LBP risk.

The rationale for conducting the current systematic review and meta-analysis can be found on pages 2-3 (see the introduction).

1. **The contribution that the systematic review makes to knowledge in light of previously published related reports, including other meta-analyses and systematic reviews**

To our knowledge, this is the first review to investigate the quantitative risk of LBP associated with different amounts of sedentary behavior. The results of this review revealed that the sedentary duration exceeding 2, 4 or 6 h/d was associated with a lower risk of LBP. However, this association disappeared when the analysis was restricted to sedentary duration exceeding 8 h/d.

See the last paragraph of the introduction (page 4), and the discussion and conclusion sections for information about the contribution of the current systematic review.
